# Beyond Photochromism: Alternative Stimuli to Trigger Diarylethenes Switching

**DOI:** 10.1002/advs.202410524

**Published:** 2024-11-03

**Authors:** Qi Ai, Kangjun Lan, Lin Li, Zugang Liu, Xiaoguang Hu

**Affiliations:** ^1^ College of Optical and Electronic Technology China Jiliang University Hangzhou 310018 China; ^2^ School of Materials Science and Engineering Zhengzhou University Zhengzhou 450001 China

**Keywords:** acidochromism, diarylethenes, electrochromism, photochromism, thermochromism

## Abstract

Diarylethenes (DAEs) are typical photochemically reversible type (P‐type) photochromic materials with excellent thermal stability and high fatigue resistance and are widely exploited as photo‐switches for various applications in molecular devices, data storage, photoresponsive materials, and bioimaging, etc. In recent years, there is an increasing number of reports using heat, acid, electrochemistry, etc. to drive the isomerization reaction of DAEs. The response to two or more different stimuli enables multi‐functionality within a single DAE molecule, which would facilitate complex logic‐gate operations, multimode data storage, and increased information density. Herein, the recent advances in DAE systems utilizing stimuli “beyond photo” to trigger the isomerization processes from three perspectives: acidochromism, thermochromism, and electrochromism are reviewed. Emphasis is placed on the molecule design strategies and the underlying mechanisms for cyclization and cycloreversion processes addressed by the alternative stimulus. Then the noticeable applications made in multi‐stimuli responsive DAE systems are summarized. Additionally, the challenges and opportunities of DAE switches driven by stimuli “beyond photo” in the future are also discussed.

## Graphical abstract

This review summarizes the recent progress of diarylethenes utilizing beyond‐photo stimuli to trigger the isomerization reaction from three perspectives: acidochromism, thermochromism, and electrochromism. The molecule design strategies and mechanism behind the cyclization and cycloreversion processes are discussed. The related applications are stated. Challenges and opportunities for the future development are also presented.

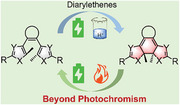

## 1 Introduction

**Figure 1:**
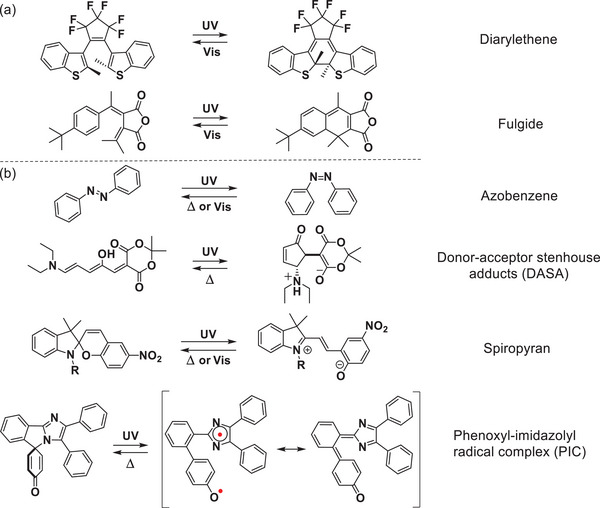
Structures of typical a) P‐type photochromic materials: DAEs and fulgide; b) T‐type photochromic materials: azobenzene, DASA, spiropyran, and PIC.

Photochromism is the phenomenon where light stimuli induce reversible interconversion between isomeric structures with distinct optical, electronic, geometrical, magnetic, coordination properties, dipole interaction, refraction coefficient, and dielectric constant as well. This phenomenon is regarded as one of the representative molecular switching events. To date, numerous photochromic materials have been developed, which can be mainly classified into P‐type and thermally reversible type (T‐type) (**Figure** **1**). The former are thermally stable species including diarylethenes (DAEs)^[^ ^1^, ^2^ ^]^ and fulgide.^[^ ^3^, ^4^ ^]^ In contrast, T‐type families are thermally reversible, such as azobenzene,^[^ ^5^, ^6^ ^]^ donor‐acceptor Stenhouse adducts (DASA),^[^ ^7^, ^8^ ^]^ spiropyran,^[^ ^9^, ^10^ ^]^ and phenoxyl‐imidazolyl radical complex (PIC),^[^ ^11^, ^12^ ^]^ etc. Recent advancements have witnessed the significantly improved photochromic performance of such materials, and their promising applications as photoswitches, photo‐controllable materials, and molecular devices.

Among these photochromic families, DAEs have been considered a milestone in the photochromic realm since their discovery in 1988 by Irie and colleagues.^[^ ^13^ ^]^ As one of the typical P‐type photochromic materials, DAEs exhibit excellent thermal stability, fatigue resistance, high‐speed photoresponse, and outstanding performances in both solution and solid states.^[^ ^2^, ^14^, ^15^ ^]^ Surprisingly, recent investigation reveals that the replacement of light by alternative external stimuli such as chemicals, heat, or electrochemistry can also trigger the isomerization reactions of DAEs.^[^ ^16^, ^17^, ^18^ ^]^ Such capability responding to two or more different inputs, enriches the functions of DAEs and endows multifunctionality within a single molecule, which would facilitate complex logic‐gate operations, multimode data storage, and increased information density and security.^[^ ^19^ ^]^ The development of photochromic DAEs that respond to beyond‐light stimuli has become an important area of research, and great progress has already been made in the switching mechanism, applications in sensing, as well as information processing and storage. Nowadays there is a solid basis of fundamental understanding with regard to the design of DAEs on the molecular level. According to the current reports, the strategies for alternative stimuli beyond light to address DAE switching can be classified into three categories: 1) acidochromism, 2) thermochromism, and 3) electrochromism. Among them, acid can convert open isomers of DAEs into closed‐ring isomers, and heat can transform closed‐ring isomers into open form. While electrochemistry is capable of driving both ring close and open processes.

The photochromism of DAE has been well‐established, and the related properties, molecular design, mechanism, and application have already been systematically reviewed.^[^ ^15^, ^20^, ^21^, ^22^, ^23^ ^]^ However, a comprehensive review of alternative stimuli to address DAEs switching is not found although great progress has been made over the past decades. As a consequence, it is the right time to summarize the current development of alternative stimuli beyond light for DAEs. Herein, this review aims to provide a summary of the typical design strategies and recent achievements made in beyond‐light stimuli switching DAE systems and reveal the underlying mechanisms for cyclization and cycloreversion in these systems. In addition, the review will present a discussion on the future major challenges, as well as opportunities anticipated in this field.

## 2 Alternative Stimuli to Switch DAEs

### 2.1 Acidochromism

**Figure 2:**
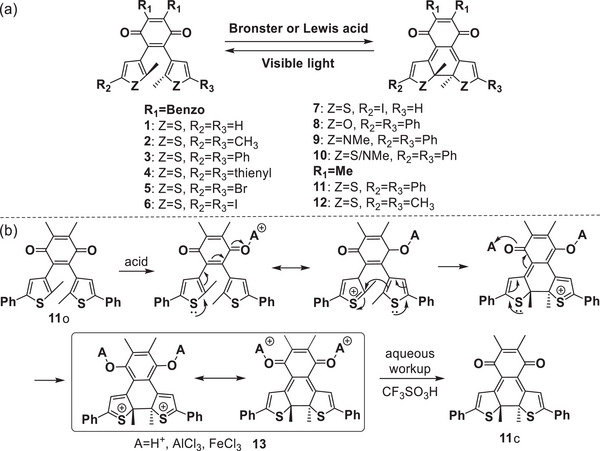
a) Molecular structures of DAEs **1‐12**; b) proposed mechanism of acid‐induced cyclization of DAE **11**.

Starting from the hypothesis that the incorporation of a quinone moiety into the molecular switch could endow it with novel redox and polar characteristics during the switching process. Liebeskind et al.,^[^ ^16^ ^]^ Tsuda and coworkers^[^ ^24^, ^25^ ^]^ successively developed a series of novel DAEs **1**–**12** (**Figure** **2**a) based on 2,3‐bis(heteroaryl)quinone core. Surprising, it was found that these photo‐switches can efficiently transform into closed‐ring isomers from open structure in the presence of strong protic or Lewis acids such as AlCl_3_, FeCl_3_, and CF_3_SO_3_H, yielding high conversion yields (≈75–95%). Upon visible light irradiation, the open‐ring isomers were recovered, along with the disappeared closed structures. Figure 2b illustrates a plausible mechanism for the acid‐induced conversion of the open structure to the closed‐ring isomer of molecules **1**–**12**. Taking DAE **11** as an example, when treated with acid, the oxygen of quinone coordinated with cation, resulting in sulfonium salt intermediate, which was then attacked by the active carbon of thiophene, and a closed structure formed. Consequently, the other quinone complexed with acid, generating the bis‐sulfonium salt intermediate **13**, and after aqueous workup or treated with Et_3_N the closed isomer **11**c formed. Therefore, the acid‐induced cyclization reaction requires at least 2 equivalents of protic or Lewis acid. To confirm the reaction mechanism, the open isomer of **11** in CD_2_Cl_2_ was treated with CF_3_SO_3_H, and the stable intermediate **13** was monitored by ^1^H and ^13^C NMR spectra, which matched the stoichiometric details of the reaction and supported the above‐proposed mechanism. Considering the indispensable quinone moiety in the acid‐induced cyclization reaction, it is supposed that the incorporation of quinone into the side position of armed thiophene may also endow DAEs with acid‐triggered cyclization characteristics. On the other hand, the replacement of thiophene with benzothiophene may also work.

### 2.2 Thermochromism

**Figure 3:**
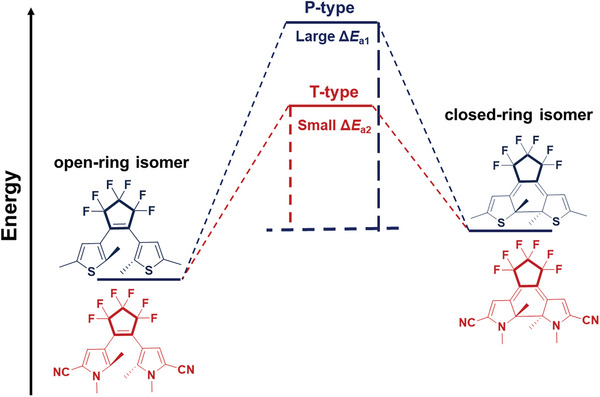
Energy diagram for the P‐type and T‐type DAEs.

According to the Woodward‐Hoffmann rule, the cycloreversion of DAEs undergoing a 6π electrocyclic reaction between cyclohexadiene and hexatriene skeletons should follow a conrotatory pathway upon illumination with visible light, preserving the symmetry of the molecular orbitals before and after the reaction.^[^ ^26^ ^]^ DAEs with thiophene and/or benzothiophene as the side aryls and perfluorocyclopentene as the ethene bridge are known for their excellent thermal stability in both open‐ and closed‐ring isomers, which are generally classified as P‐type photochromic molecules (**Figure** **3**). However, thermal cycloreversion proceeded via a disrotatory pathway was experimentally observed, which is forbidden by the Woodward‐Hoffmann rule.^[^ ^27^ ^]^ Generally, a large activation energy (*E*_a_) in the ground state means high thermal stability of the closed‐ring isomer, thereby, the value of *E*_a_ is the key factor of thermostability. Up to now, various chemical modification methods, such as altering side aryls, the ethene bridge, and reactive carbons, have been reported to modulate the *E*_a_ to achieve thermal addressing switching of DAEs.^[^ ^17^, ^27^, ^28^ ^]^ In order to transform P‐type DAEs into T‐type, the following alterations can be considered: 1) the aromatic stabilization energy of the aryl groups; 2) electron‐withdrawing substituents in the aryl groups; 3) the aromaticity of the central ethene bridge; and 4) steric hindrance of the substituents at reactive positions.

**Figure 4:**
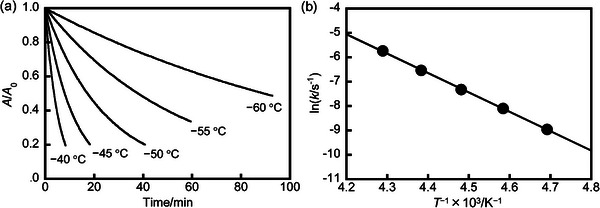
a) Absorption decay curves of DAE monitored at a specific wavelength in solution at various temperatures; Reproduced with permission.^[^ ^17^ ^]^ Copyright 2019, Royal Society of Chemistry. b) Temperature dependence of the rate constant *k* for thermal cycloreversion of DAE. Reproduced with permission.^[^ ^17^ ^]^ Copyright 2019, Royal Society of Chemistry.

The activation energy is a crucial parameter for evaluating whether a thermal cycloreversion can occur or not, and usually, the activation energy between the closed and open‐ring isomers in the ground state is very low if the thermal cycloreversion can take place. Here, we also summarize the methods for calculating activation energy as described in the referenced literature.^[^ ^17^ ^]^ Generally, the thermal cycloreversion reaction from the closed to open form obeys first‐order kinetics, the kinetic equation can be expressed as follows by using the Lambert‐Beer law (**Figure** **4**a).

(1) $$\ln\frac{A_{t}}{A_{0}}=-kt$$

where *k* is the reaction rate constant, *t* is reaction time, and *A*_0_ and *A*_t_ are the absorbance of the closed‐ring isomer at the initial state (*t* = 0 s) and at arbitrary reaction time *t*, respectively. The *k* value can be calculated from the slope of the linear plot.

Arrhenius's equation can be described as follows.

(2) $$\ln k=\ln A\;-\frac{E_{a}}{RT}$$

where *A* is the frequency factor, *E*_a_ is the activation energy, R is the gas constant, and *T* is the absolute temperature. The linear relationship can be obtained by plotting ln *k* relative to 1/*T* (Figure 4b). Thus, the *E*_a_ and *A* values can be determined from the slope and intercept of the linear plot. Generally, an activation energy barrier of ≈20 kcal mol^−1^ (84 kJ mol^−1^) is typically used as the criterion to determine whether a first‐order reaction is likely to occur readily at room temperature.^[^ ^29^ ^]^

#### 2.2.1 Modification of the Side Aryls

**Figure 5:**
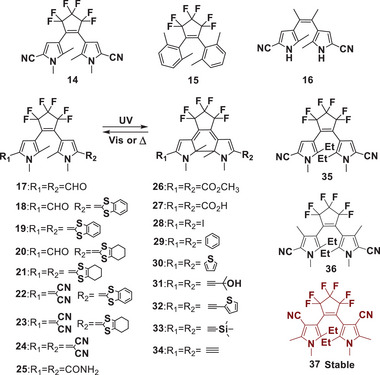
Molecular structures of DAEs **14**‐**37**.

Generally, the disrotatory thermal cycloreversion reaction of DAEs corresponds to the thermal stability of the closed‐ring isomers. When aryl groups such as pyrrole, indole, or phenyl groups are introduced as side aryls, the DAEs undergo thermally reversible photochromic reactions, due to the higher aromatic stabilization energies of these aryl groups in comparison with thiophene or benzothiophene.^[^ ^28^ ^]^ This is the most commonly used strategy for designing thermally reversible DAEs. Irie et al.^[^ ^28^ ^]^ reported several DAEs, including the dipyrrolyl‐perfluorocyclopentene (DPC) derivative **14**, to establish a guiding principle for the molecular design of thermally reversible photochromic compounds. The combination of a perfluorocyclopentene ring as the ethene moiety and 5‐cyano‐2‐methyl‐3‐pyrrolyl rings as the aryl groups in **14** significantly increased the thermal back reaction rate. The half‐life times are 68 s at 284 K and 121 s at 274 K, which is much faster than those of their previously reported 1,2‐bis(2,6‐dimethyl phenyl)perfluorocyclopentene **15** ^[^ ^30^ ^]^ and 2,3‐bis(5‐cyano‐2‐methyl‐3‐pyrrolyl)‐2‐butene **16**,^[^ ^31^ ^]^ whose half‐life time for cycle reversion at room temperature were 15 and 32 min, respectively. To systematically study the thermal stability of DPCs, Samat et al.^[^ ^32^, ^33^ ^]^ developed a series of new derivatives (**17**‐**34**) with variable π‐conjugated chain lengths (**Figure** **5**). The closed‐ring isomers of these compounds are thermally unstable, and their coloration decay is highly dependent on the nature of the substituents. Kinetic and thermodynamic study of thermal bleaching revealed that the half‐life of the cycloreversion process ranged from 6 s to 6000 s, varying by ≈10^3^ times. Notably, the most stable closed‐ring isomers, with phenyl (**29**) or ethynyl (**32** and **34**) substituents, have half‐lives of 5400, 4350, and 5991 s, respectively.

In 2019, further research by Irie et al.^[^ ^34^ ^]^ focused on DPC derivatives **35**–**37**, which featured ethyl groups at the 2‐ and 2′‐positions (**35**), methyl substituents at 4‐ and 4′‐positions (**36**), and cyan at 4‐ and 4′‐positions (**37**) of the pyrrole rings. The half‐life of the closed‐ring isomer of **35** is ≈0.4 s, significantly shorter than that of DAE **14**. A decrease in the thermal fading rate was observed for DAE **36**, while no noticeable decrease in the absorption spectrum was observed at ambient temperature for **37**. It is concluded that the thermal stability of the closed‐ring isomer of DPC derivatives depends on the alkyl substituents at the 2‐ and 2′‐positions, and the ethyl‐substituted derivative degraded much faster than that of the methyl‐substituted derivative. On the other hand, similar to cyan groups, the electron‐withdrawing groups at 5‐ and 5′‐positions can decrease the thermal stability of closed DPC derivatives, while phenyl, alkyl, and alkyne can slow down the decay rate. This difference in thermal stability was explained by the difference in the ground state energies between open‐ and closed‐ring isomers.

**Figure 6:**
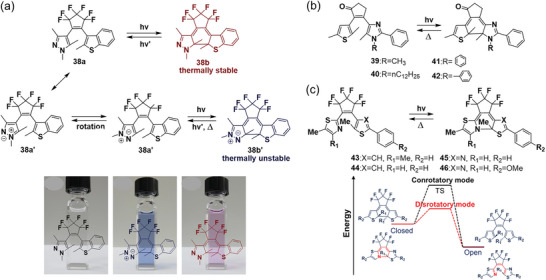
a) Proposed mechanism of the photoisomerization of **38** and the corresponding color changes in acetonitrile; reproduced with permission.^[^ ^35^ ^]^ Copyright 2019, The Chemical Society of Japan. b) Molecular structures of DAEs **39**–**42**; c) molecular structures of DAEs **43**–**46** and energy diagram for the thermal cycloreversion of common DAEs and **43**–**46**.

If DAEs exhibit both P‐type and T‐type photochromism, they can be developed into novel multistate/multifunctional materials. However, DAEs showing simultaneous P‐ and T‐type photochromism are still rare. In 2019, Saita et al.^[^ ^35^ ^]^ introduced pyrazole and benzothiophene groups as heteroaryl groups, and the resulting DAE **38** exhibits both P‐ and T‐type photochromism. Upon UV irradiation, **38**a generated two kinds of colored isomers simultaneously, and both colored isomers could be reversibly converted to the open‐ring isomer by visible light irradiation. The pink‐colored isomer was thermally stable, while the blue isomer reverted to the initial colorless state upon heating. The possible mechanism for the photoisomerization proposed in **Figure** **6**a suggests that the pyrazole ring in an open isomer can resonate between neutral and zwitterion forms with a double bond between 3‐ and 4‐positions. Then the resulting **38**a′ rotated and cyclized to closed‐ring isomer **38**b′ under UV light illumination. It should be noted that **38**a and **38**a′ exist in equilibrium due to the rapid rotation of the pyrazole unit. The thermal stability of the closed‐ring isomers was examined in acetonitrile, and the half‐life time of **38**b′ was estimated to be 50 min at 25 °C and 3.2 min at 70 °C. The rate constant of the thermal cycloreversion of **38**b′ was determined from the slope of the first‐order plots, and the activation energy (*E*_a_) and frequency factor (A) can be estimated from the Arrhenius equation with values estimated to be 52 kJ mol^−1^ and 3.1 × 10^5^ s^−1^, respectively.

On the other hand, Shirinian et al.^[^ ^36^ ^]^ reported that the utilization of imidazole as a side aryl group (DAEs **39**–**42** in Figure 6b) can improve the thermal stability compared to that of pyrrole and pyrazole cases. Thermal stability investigations were conducted in acetonitrile at 293 K in the dark, and the half‐life of the closed **39** and **40** is estimated to be ≈30 h. Moreover, the substituent of phenyl or benzyl at N can further improve the thermal stability, the half‐life of closed **41** and **42** was calculated to be 175.0 and 61.1 h, respectively. This modification also improved both the cyclization and cycloreversion quantum yields and provided a valuable route to tune the thermal stability of DAEs.

Kobatake et al.^[^ ^37^ ^]^ discovered that thermal cycloreversion can also follow the Woodward‐Hoffmann rule. DAEs **43**–**46** with thiazole derivatives as the aryl group were developed to investigate their photochromic behaviors based on 6π aza electrocyclic reactions (Figure 6c). Upon UV irradiation, a new absorption band ≈500 nm in the visible light region was observed in all cases. When UV light was removed, the visible absorption bands disappeared even in the dark, and the absorption spectra returned to the initial state. DAEs **43**–**46** exhibited fast T‐type photochromism with the half‐life (t_1/2_) ranging from µs to ms, and at 298 K t_1/2_ of the closed‐ring isomers was calculated to be 0.18, 7.18, 13.2, and 27.6 ms, respectively. The experimental activation energy *E*_a_ values for **43**–**46** were 58.8, 62.5, 63.3, and 65.1 kJ mol^−1^, respectively. To elucidate the reaction mechanism, they analyzed the molecular geometry changes during the thermal cycloreversion reaction using IRC calculations. They found that DAEs **43**–**46** follow a disrotatory pathway, whereas common diarylethenes undergo a conrotatory pathway due to the presence of substituents, such as methyl groups, at the reactive carbons, which block the disrotatory pathway allowed by the Woodward‐Hoffmann rule. Consequently, their closed‐ring isomers can only proceed via the conrotatory cycloreversion, which requires high activation energy and results in thermal stability. In contrast, DAEs **43**–**46** lack substituents at the nitrogen atom at the reaction site (Figure 6c). The lone pair on the nitrogen, not fixed due to the low pyramidal inversion barrier energy of amines, enables the disrotatory pathway with low activation energy. These results provide useful information for the dynamics of the 6π aza electrocyclic reaction and further development of thermochromic DAEs utilizing this reaction.

#### 2.2.2 Modification of the Ethene Bridge

**Figure 7:**
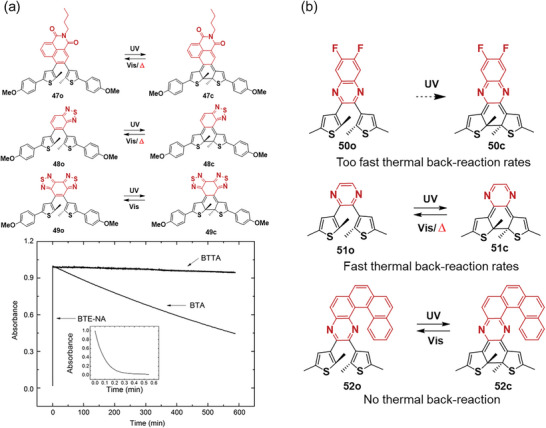
a) Molecular structures of DAEs **47**‐**49**, and the thermal back‐reaction of BTE‐NA **47**, BTA **48**, and BTTA **49** in chloroform at 293 K; reproduced with permission.^[^ ^38^ ^]^ Copyright 2012, Wiley‐VCH. b) Molecular structures of DAEs **50**‐**52**.

Several groups have investigated the thermal stability of utilizing aromatic rings as the ethene bridge unit, demonstrating that the strong aromaticity of the central ethene bridge reduces the energy barrier for cycloreversion. Zhu et al.^[^ ^38^ ^]^ designed three novel DAEs, BTE‐NA **47**, BTA **48**, and BTTA **49**, containing naphthalimide, benzothiadiazole, and benzobisthiadiazole as ethene bridges with varying aromaticities (**Figure** **7**a). Among them, **47** and **48** displayed thermal cycloreversion, and the thermal back‐reaction rates for closed isomers depended on the solvents due to the activation energy difference in various solvents. The thermal back‐reaction rate for **48**c (t_1/2_ = 4.58 × 10^4^ s) was four orders of magnitudes slower than that of **47**c (t_1/2_ = 3.89 s) due to decreased aromaticity in the ethene bridge. While **49**, the control molecule with the weakest aromaticity in the ethene bridge was thermal stable and did not show thermochromism. This work enhances the understanding of aromaticity‐controlled thermal stability of photochromic systems using a six‐membered ring as the ethene bridge. Diederich and coworkers^[^ ^39^ ^]^ provided the first example of DAE systems **50**–**52** with 6π heteroaromatic six‐membered ring bridge (Figure 7b). It was found that pyrazine functionalized **51** was thermally unstable and the closed isomer underwent a cycloreversion with a half‐life (t_1/2_) of 69 s in the absence of light. Surprisingly, irradiation of quinoxaline containing DAE **50** did not result in any observable changes in the absorption spectra, suggesting that the photocyclization reaction is suppressed, which may be due to the loss of aromaticity upon cyclization and stabilization of the twisted intermolecular charge transfer state. Conversely, further π‐extension by fusing phenanthrene yielded thermally irreversible DAE **52**, the closed form in toluene did not show any observable thermal back‐reaction because it retains the diatropic currents within the adjacent aromatic rings.

**Figure 8:**
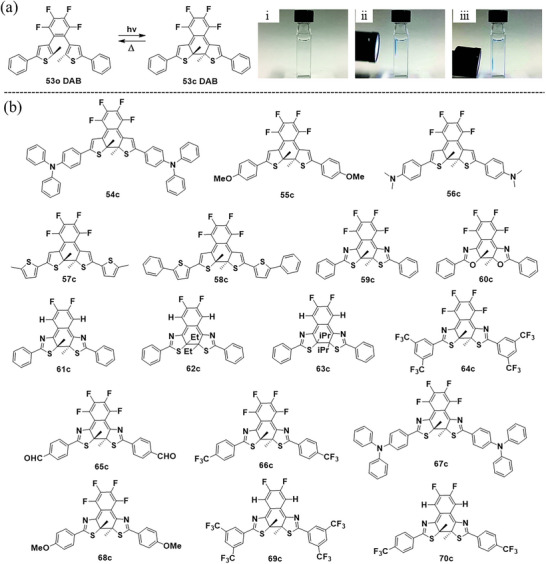
a) Molecular structures of DAB **53**, and (i) before photoirradiation and upon irradiation with 313 nm light to (ii) the upper part and (iii) the lower part of the quartz cuvette; Reproduced with permission.^[^ ^17^ ^]^ Copyright 2019, Royal Society of Chemistry. b) Molecular structures of close‐ring isomers of DAB derivatives **54**‐**70**.

Subsequently, based on a membered ethene bridge, Kobatake et al. have developed a series of thermally reversible DAEs (**Figure** **8**). In 2019, they^[^ ^17^ ^]^ presented a photochromic 1,2‐diarylbenzene (DAB **53**) with fast thermal cycloreversion (t_1/2_ = 0.13 s) by incorporating a tetrafluorophenyl ring as ethene bridge and 2‐methyl‐5‐phenylthiophene as aryl groups. Introducing electron‐donating groups^[^ ^40^ ^]^ such as *N, N*‐diphenylamine (**54**), methoxy (**55**), or *N*, *N*‐dimethylamino (**56**) to the DAB parent molecule resulted in a red‐shift in the absorption spectra, an increase in the absorption coefficients of the open‐ring isomers, and deceleration of the thermal cycloreversion (t_1/2_ = 0.33–1.14 s) of the closed‐ring isomers as well. To enhance the photosensitivity in the UV‐A region without altering the thermal back‐reaction rate, the phenyl at 5‐position of thiophene was replaced with thiophene derivatives. The resulting molecules **57** and **58** ^[^ ^41^ ^]^ displayed increased oscillator strength and absorption coefficient, and improved photosensitivity in the UV‐A region without changing the thermal back‐reaction rate (t_1/2_ = 0.09–0.28 s).

It was found that the thermochromism of DAB derivatives is not affected when the five‐membered heteroaromatic side groups are changed as oxazole (**59**) and thiazole (**60**).^[^ ^42^ ^]^ To achieve a fast T‐type photochromic reaction in the crystalline state, in 2021, a rationally designed molecule **61** ^[^ ^43^ ^]^ was realized to stabilize the antiparallel conformation with a short reactive carbon distance by forming intramolecular CH─N hydrogen bonding between the hydrogen atom of the ethene bridge and the nitrogen atom of the thiazole rings. In the same year, nanoparticles^[^ ^44^ ^]^ were fabricated from **61** using ball‐milling and reprecipitation methods. The ball‐milled nanoparticles exhibited crystallinity, while the reprecipitated nanoparticles were amorphous, both displaying reversible T‐type photochromic behavior. Thus, T‐type photochromic crystals and nanoparticles were successfully developed based on **61**. Furthermore, bulky substituents at the reactive carbons can accelerate the thermal back reaction rate. DABs **62** and **63**,^[^ ^45^ ^]^ which have ethyl and isopropyl groups at the reactive carbons instead of methyl groups, showed faster coloration (t_1/2_ = 0.54–30 s) compared to **61** (t_1/2_ = 280 s). Recently, electronic effects on the thermal back reaction were studied by modifying compounds **59** and **61** with electron‐donating and withdrawing groups, i.e., **64–70**. Their experimentally obtained activation energy for the thermal back reaction was accurately reproduced by DFT calculations using the M06‐2X level and 6–31G(d) basis set. As summarized in **Table** **1**, the introduction of electron‐donating groups can lift the Δ*E*_a_ and prolong the t_1/2_. In contrast, electron‐withdrawing groups can reduce the Δ*E*_a_ and shorten the t_1/2_. Such a tendency is similar to the thiophene case, e.g., **53**–**56**. These findings provide a valuable strategy for designing novel DAEs with desired thermal back reactivity.^[^ ^46^ ^]^

**Table 1:** Thermally switching DAB derivatives and their corresponding activation energies (Δ*E*_a_/ kJ·mol^−1^) and lifetimes t_1/2_.

Molecules	Δ*E*_a_	t_1/2_/s	Molecules	Δ*E*_a_	t_1/2_/s
**53**c	66	0.13	**62**c	86	30
**54**c	69	0.33	**63**c	68	0.54
**55**c	71	0.51	**64**c	65	0.10
**56**c	77	1.14	**65**c	65	0.13
**57**c	67	0.28	**66**c	69	0.25
**58**c	63	0.09	**67**c	79	7.8
**59**c	76	1.6	**68**c	81	10
**60**c	80	580	**69**c	80	17
**61**c	88	280	**70**c	83	37

**Figure 9:**
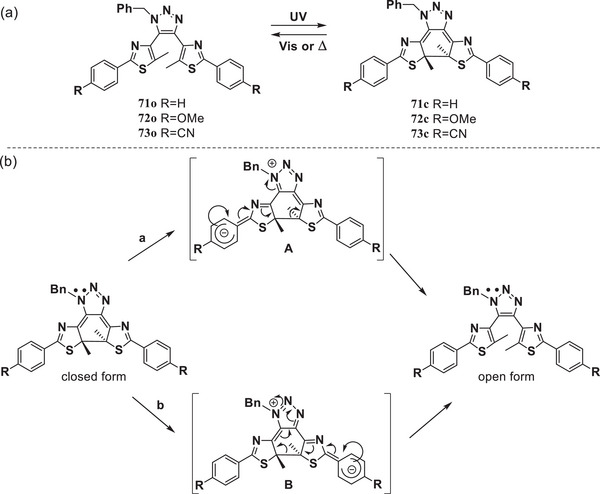
a) Molecular structures of DAEs **71**‐**73**; b) The proposed mechanism of thermal cycloreversion.

Yokoyama et al.^[^ ^47^ ^]^ employed a “click” reaction to introduce a five‐membered triazole ring as the ethene bridge to construct DAEs **71**–**73**. All closed‐ring isomers are thermally reversible, the thermal back reaction of **73**c containing electron‐withdrawing cyano groups was the fastest (t_1/2_ = 11.7 s). While that of **72**c, with electron‐donating methoxy groups, was the slowest (t_1/2_ = 1130 s). The proposed mechanism for the acceleration of thermal cycloreversion by electron‐withdrawing groups is illustrated in **Figure** **9**b. When the delocalized lone pair on the nitrogen atom moves back from the resonance structures A and B, it may break the central C─C single bond to revert to the open form, as shown by the arrows. The resonance structures are more strongly stabilized when R is a cyano group, facilitating the scission of the C─C bond. Therefore, the rate of the thermal cycloreversion can be controlled by 1) changing the aromatic ring at the ethene moiety and 2) modifying the substituents on the phenyl rings at the periphery of the molecule.

#### 2.2.3 Modification of the Reactive Carbon Atoms

**Figure 10:**
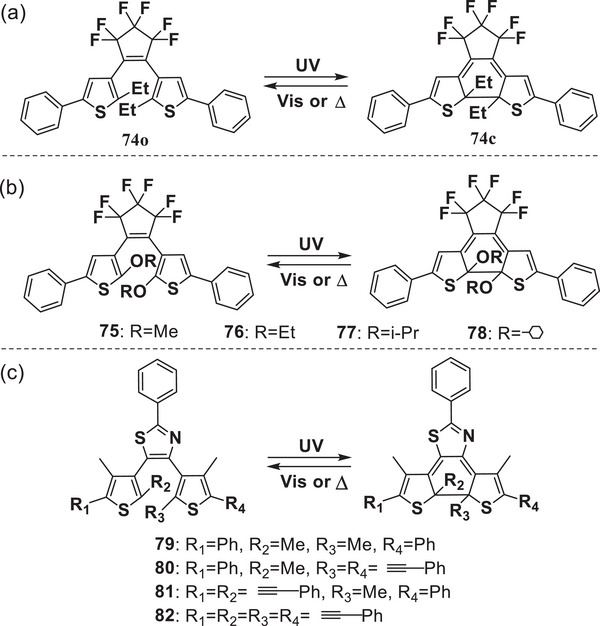
Molecular structures of DAEs **74**‐**82**.

Bulky substituents at the active carbons induce strain in the molecular structure of the closed‐ring isomers, resulting in decreased activation energy.^[^ ^1^ ^]^ DAE **74**,^[^ ^27^ ^]^ with ethyl substituents at 2‐ and 2′‐ positions of the thiophene rings, undergoes a thermally reversible cycloreversion reaction (**Figure** **10**a). The blue closed isomer **74**c in solution and single crystal returns to a colorless form **74**o upon irradiation with visible light or heating above 100 °C. The thermal cycloreversion activation energies from **74**c to **74**o were measured to be 128 kJ mol^−1^ in toluene solution and 137 kJ mol^−1^ in a single‐crystalline phase above 100 °C. Remarkably, the thermal cycloreversion in the crystal was directly observed by X‐ray crystallography in a conrotatory mode. Irie's group prepared dithienylethene derivatives **75**–**78** ^[^ ^48^ ^]^ with various alkoxy substituents, such as ethoxy, isopropoxy, and cyclohexyloxy, at the reactive 2‐and 2′‐ positions of the thiophene (Figure 10b), because bulky alkoxy groups are known to enhance thermal decoloration reactions at high temperatures. The half‐life times of **75**c, **76**c, **77**c, and **78**c at 160 °C were 18 min, 7.4 min, 1.6 min, and 45 s, respectively. Notably, **78**c displayed fast thermal cycloreversion at high temperatures but thermal stability at room temperature. Subsequently, Kawai et al. synthesized 4,5‐dithienylthiazole‐based DAEs **79**–**82** ^[^ ^49^ ^]^ (Figure 10c) with high coloration reactivity, and their thermal cycloreversion time constant can be modulated over 10^5^ times by incorporating phenylethynyl groups. In particular, the four phenylethynyl‐containing DAE **82** showed rapid bleaching in darkness at 293 K (<10 s) and less than 1 s at 313 K even within a polymer matrix.

### 2.3 Electronchromism

**Figure 11:**
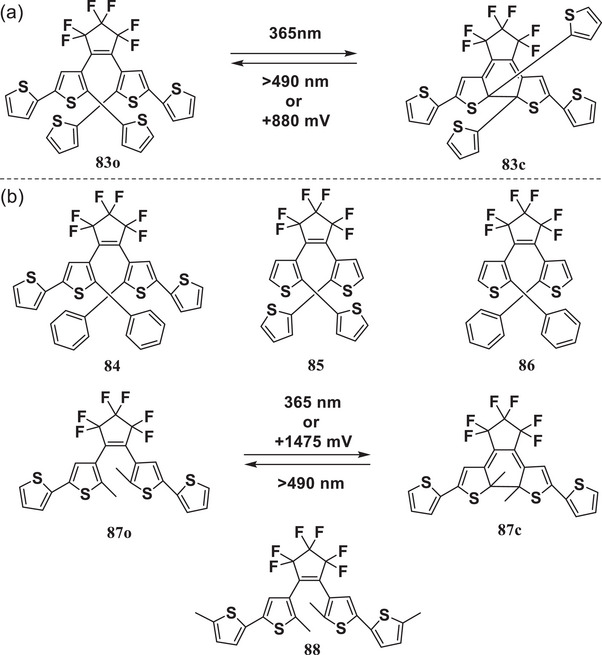
Pioneering works on electrochemical triggered a) cycloreversion and b) cyclization.

In 2003, Branda et al.^[^ ^18^ ^]^ observed unprecedented electrochromic properties in a class of DAEs (**83**‐**86**, **Figure** **11**a) with either thiophene or phenyl groups attached to the reactive carbons of 1,2‐dithienylalkene derivatives. In addition to photochromism, these compounds can be catalytically transformed from ring‐closed states to ring‐open states through electrochemical or chemical oxidation. This represents the first example of a 1,2‐dithienylalkene derivative exhibiting both photochromic and electrochromic ring‐opening. In the same year, an unprecedented cyclization triggered by electrochemical oxidation was observed for DAEs **87** and **88** ^[^ ^50^ ^]^ (Figure 11b). These findings initiated the investigation of electrochromic DAEs. Subsequently, the electrochromism of DAEs became a research subject of intense interest, and the exact structure‐property relationships, limitations, requirements, and mechanisms were gradually uncovered. Currently, numerous DAEs including transition‐metal‐containing complexes or with various substituents in central bridging units or side aryls, have been designed with diverse electrochemical responses for electrochromism.

#### 2.3.1 Electrochromic Switching Mechanism

In contrast to well‐established photochromism, the electrochromic mechanisms were relatively clear. Two general mechanisms have been proposed. The Branda,^[^ ^18^ ^]^ Launay,^[^ ^51^ ^]^ Irie,^[^ ^52^ ^]^ and Kawai^[^ ^53^ ^]^ groups identified that radical cations are responsible for oxidative ring opening or closing reactions, while the Feringa,^[^ ^54^ ^]^ Yu,^[^ ^55^ ^]^ and Akita^[^ ^56^ ^]^ groups suggested that the reactions proceed via dicationic states.

**Figure 12:**
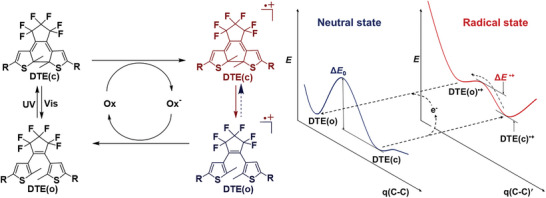
The proposed electrochemical mechanism for cycloreversion via radical cation states.

Taking the radical cation cycloreversion mechanism of dithienylethene (DTE) as an example, a radical cation DTE(c)^•+^ was generated instantaneously when one electron oxidant was added to DTE(c). This radical cation is easily converted to DTE(o)^•+^ due to significantly lower barrier energies compared to the energy barrier from DTE(c) to DTE(o) (i.e., Δ*E* ^•+^ < Δ*E*_0_ in **Figure** **12**). Subsequently, DTE(o)^•+^ was reduced to DTE(o), which can act as the oxidant to oxidize DTE(c) to DTE(c)^•+^. Because the energy gap between DTE(o)^•+^ and DTE(o) is larger than that between DTE(c)^•+^ and DTE(c), the cycloreversion proceeds as a chain reaction. With the aid of an electron transfer stopped‐flow method, several research groups^[^ ^57^, ^58^ ^]^ spectroscopically observed both intermediates, DTE(c)^•+^ and DTE(o)^•+^, further supporting the mechanism.

**Figure 13:**
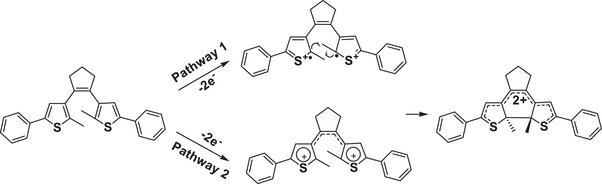
The electrochemical mechanism for cyclization via a dicationic state.

For the dicationic involved cyclization mechanism (**Figure** **13**), starting from DTE(o), oxidation at a relatively high voltage directly forms the dication DTE(o)^2+^. Then delocalization of the charge over the entire conjugated system drives the cyclization of DTE(o)^2+^ to DTE(c)^2+^. There are two possible pathways for response to this process. Pathway one involves α‐coupling at the α‐positions to form a carbon‐carbon single bond, probably leading to both *trans* and *cis* DTE(c)^2+^. However, steric factors favor the formation of the same product (after reduction) as the photochemical cyclization. The alternative pathway is a 4‐π electron cyclization that proceeds thermally in a disrotatory manner, which follows the Woodward‐Hoffmann rule and is analogous to the photochemical reaction. In a word, either pathway leads to the formation of closed‐ring dication.

#### 2.3.2 Organometallics

**Figure 14:**
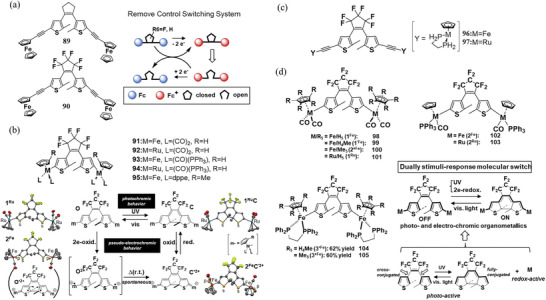
a) Molecular structures of DAEs **89**, **90**, and the mechanism of electrochromism; REPRODUCED with permission.^[^ ^60^ ^]^ Copyright 2007, American Chemical Society. b) Molecular structures of DAEs **91**–**95** and the mechanism of electrochromism; reproduced with permission.^[^ ^61^ ^]^ Copyright 2008, Royal Society of Chemistry. c) Molecular structures of DAEs **96** and **97**; d) molecular structures and design strategy of DAEs **98**–**105**. Reproduced with permission.^[^ ^62^ ^]^ Copyright 2011, Royal Society of Chemistry.

Combining chromic systems with other chemical systems, such as metal fragments with redox and catalytic functions, can lead to more sophisticated systems. Integrating chromic phenomena induced by different stimuli (e.g., photons, electricity) into a single molecule can create multi‐stimuli‐responsive systems.^[^ ^59^ ^]^ Launay et al.^[^ ^60^ ^]^ prepared perhydro‐DTE Fc‐PCH‐Fc **89** and perfluoro‐DTE Fc‐PCF‐Fc **90** with ferrocenyl substituents (**Figure** **14**a) to induce a change in chromic properties via an intramolecular electron‐transfer reaction from the redox group to the photochromic core. For the closed‐ring isomers, an electrocatalytic ring‐opening process was observed for **89**, and a slightly slower electrochemical process was found for **90**. The redox status of the ferrocenyl moieties triggers the photochromic reactivity, acting as an “antenna” that temporarily stores a charge and facilitates the cycloreversion transformation.

Soon afterward, Akita et al.^[^ ^61^ ^]^ incorporated a series of redox‐active organometallics into DTE via σ‐bond (Figure 14b), i.e., Fe/Ru(η^5^‐C_5_H_4_R)(CO)_2_ (**91** and **92**), Fe/RuCp(CO)(PPh_3_) (**93** and **94**), and FeCpʹ(dppe) **95**. Compounds **91**–**94** exhibit photochromic properties, while **95** do not undergo photochemical cyclization. However, upon oxidation, all DAEs cyclized quantitatively. In the cases of **93** and **95**, the deep‐green, dicationic species **93c**″**^2+^** and **95c**″**^2+^** were isolated upon chemical oxidation with [FeCp_2_]PF_6_ (2 equiv.). As depicted in Figure 14b and X‐ray crystallography of **93c**″**^2+^** revealed: i) C─C bond formation between the two thiophene rings at the 2‐ and 2′‐positions, ii) the double bond character of the Fe═C moieties, and iii) the change in the bond alternation pattern consistent with the canonical form **c**″**^2+^**. Since the closed species **c'^2+^** are stable under reduction or irradiation conditions, they do not undergo cycloreversion. Thus, the closed structure, otherwise prone to open even under weak daylight, can be firmly ‘‘‘locked’’’ by the electrochemical method, making this system a pseudo‐electrochromic. Additionally, dinuclear acetylide‐type complexes bridged by a photochromic DTE unit, [Y‐C≡C‐DTE‐C≡C‐Y] (**96** and **97**, Y = {MCp*(dppe)}; Cp* = pentamethylcyclopentadienyl, M = Fe (**96**), Ru (**97**))^[^ ^56^ ^]^ (Figure 14c), have been prepared. These complexes exhibit typical photochromic behavior, but performance depends on the present metal. Furthermore, the ruthenium complex **97**o undergoes oxidative ring closure to form the dicationic species of the closed‐ring isomer **97**c^2+^, demonstrating dual photo‐ and electro‐chromism. Akita et al. defined the previously discussed DAEs as the 1st generation DTE complexes, in 2021, they designed the 2nd generation DTE complexes, i.e., ***M***‐DTE‐***M*** (**98**‐**105** in Figure 14d and ***M***: M(η^5^‐C_5_R_5_)L_2_; M = Fe, Ru; R = H, Me; L = CO, PPh_3_, (dppe)½).^[^ ^62^ ^]^ Electrochemical analysis of these DAEs reveals that 2e‐oxidation of the open isomer ***M***‐DTE‐***M*** o induces ring closure of the DTE moiety, forming the Fischer‐carbene‐typedicationic closed derivatives ***M***‐DTE‐***M*** c^2+^, which have a differentπ‐conjugated structure with the neutral species ***M***‐DTE‐***M*** c. Subsequent reduction of ***M***‐DTE‐***M*** c^2+^ yields the neutral closed species ***M***‐DTE‐***M*** c.

**Figure 15:**
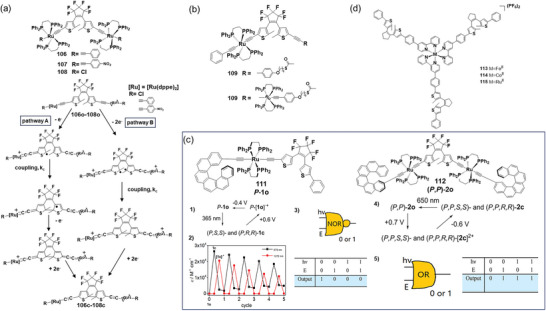
a) Molecular structures of DAEs **106**–**108**, and the mechanism of electrochromism; reproduced with permission.^[^ ^63^ ^]^ Copyright 2014, American Chemical Society. b) molecular structures of DAEs **109** and **110**; c) molecular structures of DAEs **111** and **112**, 1)‐5): three‐states cycle obtained from the system upon light and redox stimuli, the symbol of logic gates, and their truth tables; reproduced with permission.^[^ ^65^ ^]^ Copyright 2017, American Chemical Society. d) molecular structures of star‐shaped DAEs **113**–**115**.

DTEs with a ruthenium carbon‐rich system (**106**‐**108**)^[^ ^63^ ^]^ enable sophisticated and efficient light‐ and electro‐triggered multifunctional switches, featuring multicolor electrochromism and electrochemical cyclization at remarkably low voltages. The electrochemical ring closure mechanism through an intramolecular radical coupling of complexes **106**o^2+^−**108**o^2+^ (**Figure** **15**a) is thermodynamically favorable by 0.064, 0.477, and 0.026 eV, respectively. In addition to the energetic stabilization from the isomerization from **106**o^2+^−**108**o^2+^ to **106**c^2+^−**108**c^2+^ (with the energy order being **107**o^2+^≫ **106**o^2+^ > **108**o^2+^), DFT calculations indicate that **107**o is more readily electro isomerized, which is in full agreement with experimental results. Additionally, carbon‐rich ruthenium‐DTE complex **109** ^[^ ^64^ ^]^ bearing a hexylthiol spacer does not isomerize upon oxidation, while the bimetallic complex **110** can efficiently undergo electrochemical ring closure at low potential (0.5 V vs SCE) (Figure 15b). The same group then synthesized enantiomerically enriched mono‐ and bis([6]helicene‐≡‐Ru(dppe)_2_‐≡)‐DTE complexes **111** and **112**.^[^ ^65^ ^]^ The monosubstituted **111** can undergo cycloreversion from the closed oxidized state to the open oxidized state, while the bis‐substituted **112** can cyclize to the closed oxidized state by oxidating open form. These chiral complexes, exhibiting strong chiroptical responses, function as novel chiroptical switches triggered by light and/or redox stimuli and can be described as either “NOR” or “OR” logic gates (Figure 15c). For example, in the case of **111**, the open‐state **111**o, with low absorption at 679 nm, is defined as “0”, and the closed‐state **111**c, with high absorption at 679 nm, is defined as “1”. The output is measured by UV–vis absorption. Starting from **111**c, two inputs can be applied: irradiation at 650 nm and an external potential at +0.6 V, followed by −0.4 V versus Ag wire (E). These inputs are defined as either “0” and “1”. Irradiation (1,0), external potential (0,1), or both together (1,1) can trigger the cycloreversion of **111**c, forming the low‐absorption species **111**o (output 0). Thus, this process functions as a “NOR” logic gate.

Moreover, star‐shaped Fe^II^, Co^II^, and Ru^II^ tris‐bipyridine complexes (**113**–**115**) containing three DTEs, which can also be cyclized electrochemically, have been synthesized and characterized (Figure 15d).^[^ ^66^ ^]^ This research reveals that combining a photochromic system with an electrochromic system results in a more sophisticated, multi‐stimuli‐responsible system such as a dual photo‐ and electro‐chromic system, with potential applications in molecular devices. These complexes could serve as the basis for three‐terminal electronic devices by attaching anchoring groups such as pyridine, thiolate, or isocyanide. The three side arms could be bound to source, drain, and gate electrodes in a typical field‐effect transistor (FET) configuration, with the metal center in the middle of the single molecular device. Thereby, the electronic conduction through the source and drain can be modulated by the reversible and stable redox states of the metal center and/or the open/closed forms of the DTE bridges.

#### 2.3.3 DAEs Without Metals

**Figure 16:**
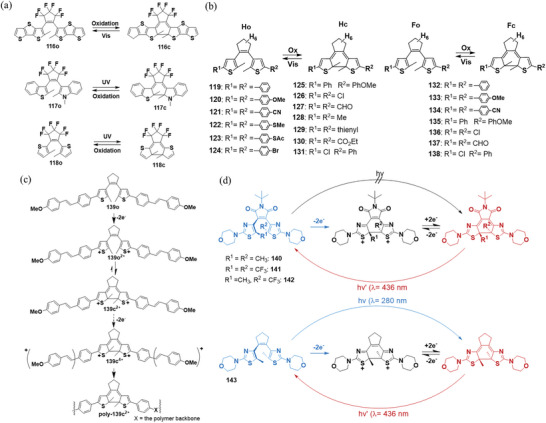
a) Molecular structures of DAEs **116**–**118**; b) molecular structures of DAEs **119**–**138**; c) sequential oxidation and ring closure steps that precede polymerization: **139**o is oxidized and forms **139**c^2+^; d) Isomerization behavior of DAEs **140**–**143**.

DAEs without metals also exhibit electrochromic properties. To elucidate the mechanism, oxidative cyclization, and cycloreversion were systematically studied, and the basic requirements for these reactions were proposed. Irie et al.^[^ ^52^ ^]^ prepared three DAEs (**116**‐**118** in **Figure** **16**a) and found that **116**o underwent oxidative cyclization, while **117**c and **118**c underwent oxidative cycloreversion. DFT calculated energies of cation radicals and neutral forms for both open‐ and the closed‐ring isomers of **116**–**118** indicate that DAEs undergo oxidative cyclization when radical cations of the closed‐ring isomers are more stable than those of open‐ring isomers. Conversely, oxidative cycloreversion occurs when the radical cations of the open‐ring isomers are more stable than those of the closed‐ring isomers.

To better understand the structure‐property relationships of electrochemically driven ring‐opening and ring‐closing of DAEs, Feringa et al.^[^ ^54^, ^67^ ^]^ studied the electronic effect on the electrochemical and spectroelectrochemical properties of a series of C5‐substituted dithienylhexahydro‐ (**119**–**131**) and dithienylhexafluoro‐ (**132**–**138**) cyclopentenes (Figure 16b). The efficiency of electrochemical switching depends on both the central cyclopentene moiety and the nature of the substituents; electron‐donating groups favor oxidative electrochemical cyclization and vice versa. The results demonstrated that the redox properties of DAEs can be tuned for either electrochemical ring‐closing or ring‐opening by stabilizing or destabilizing the mono‐ and dication of the closed state, respectively. Additionally, they introduced electropolymerisable methoxystyryl units to DAE **139** ^[^ ^68^ ^]^ and immobilized it on conducting substrates, such as gold, platinum, glassy carbon, and indium tin oxide (ITO) electrodes, and the resulting films can be switched both optically and electrochemically between the open and closed state (Figure 16c).

Hecht's group extended the aryl groups to thiazole, and the dithiazolylethene derivatives (**140**–**143**)^[^ ^69^ ^]^ undergo electrochemically driven conversion from open to closed form. The oxidative ring‐closure is facilitated by the terminal electron‐donating morpholino substituent, meanwhile, photocyclization is inhibited due to intramolecular charge transfer interaction (Figure 16d). Since cycloreversion can only be triggered by light, the system can be switched by two different “orthogonal” stimuli. Interestingly, DAE **143** bearing an electron‐neutral cyclopentene bridge showed both electrochemical and photochemical cyclization. These DAE systems could serve in multifunctional (logic) devices operated by different stimuli and may pave the way to converting light into electrical energy via photoinduced “pumping” of redox‐active metastable states.

**Figure 17:**
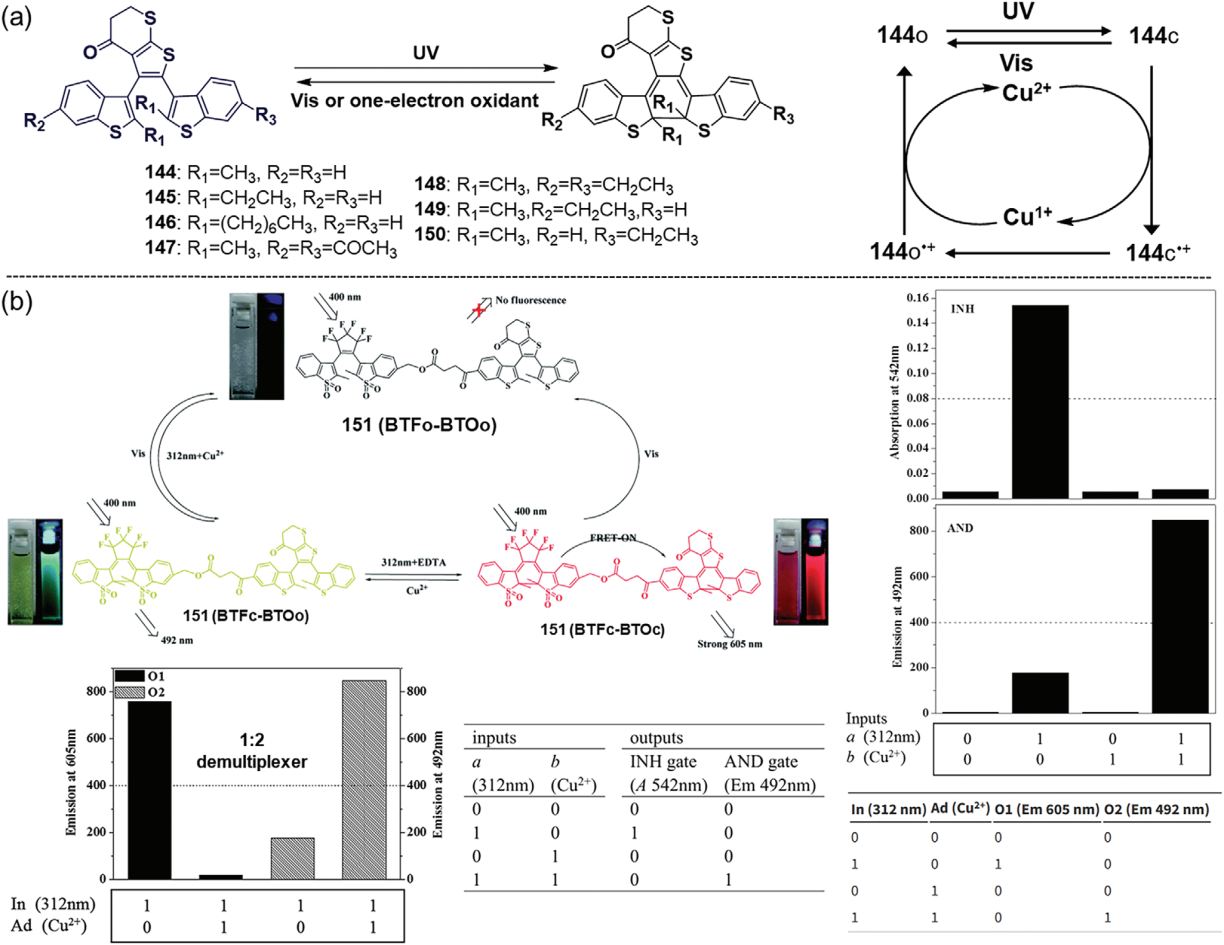
a) Molecular structures of BTHTO derivatives **144**–**150**, and the mechanism of electrochromism; b) photochemical and electrochemical interconversions among the three photoisomeric structures of **151** (BTF‐BTH), the performance of logic gates and their truth tables. Reproduced with permission.^[^ ^19^ ^]^ Copyright 2016, Royal Society of Chemistry.

Ahn et al.^[^ ^70^ ^]^ found that the fluorescence turn‐on photochromic BTHTO (**144**), featuring a 5,6‐dihydro‐4H‐thieno[2,3‐b]thiopyran‐4‐one as the ethene bridge and benzothiophene as the central hexatriene, also exhibited electrochromic property. Since **144** is electron‐rich, it was presumed to be sensitive to oxidate. As anticipated, the addition of Cu^2+^ (0.5 equiv) at the photostationary state in the dark‐induced an immediate color change from red to colorless, accompanied by the disappearance of the absorption band at 520 nm, indicating the cycloreversion of **144**c. Moreover, photoexcitation of the solution of **144**o and Cu^2+^ with 365 nm light didn't induce cyclization, revealing that Cu^2+^ completely inhibited the photocyclization of **144**, thereby acting as a specific “lock” for the open form. Interestingly, this gated behavior was reversible, quantitative titration of Cu^2+^ with EDTA restored the photoswitching behavior of **144**. Furthermore, BTHTO derivatives **145**–**150** ^[^ ^71^ ^]^ bearing alkyl and acetyl substituents showed similar electrochromic behavior (**Figure** **17**a). Our group synthesized a novel multifunctional photochromic system **151** ^[^ ^19^ ^]^ by covalently linking **148** with another fluorescence turn‐on DAE, BTFO4. Due to **148′**s electrochromic properties and the overlap between the emission of BTFO4c and absorption of **148**c, four photoisomeric states: BTFo‐BTHo, BTFc‐BTHc, BTFc‐BTHo, and BTFo‐BTHc are achievable through light irradiation and the addition of Cu^2+^ (Figure 17b). As a result, various logic gates, including INHIBIT, AND, and 1:2 demultiplexer were successfully demonstrated using light and the Cu^2+^ as inputs and fluorescence as outputs.

**Figure 18:**
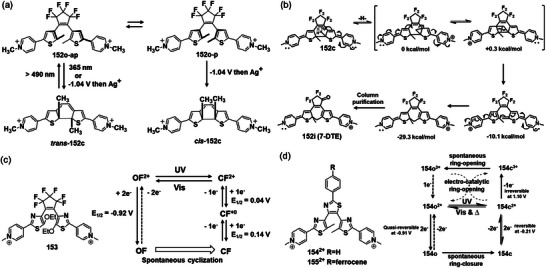
a) Photoexcitation ring closure of conformers **152**o‐ap and **152**o‐ap only produces the *trans*‐stereoisomer, while electrochemical ring‐closure can produce both *cis* and *trans* products; reproduced with permission.^[^ ^74^ ^]^ Copyright 2004, Wiley‐VCH. b) Proposed mechanism for the formation of the seven‐membered ring derivative 7‐DTE; reproduced with permission.^[^ ^77^ ^]^ Copyright 2022, Wiley‐VCH. c) Molecular structures of **153** and corresponding electrochemical transformation reaction mechanism; between 2o^2+^ and 2c^2+^; reproduced with permission.^[^ ^78^ ^]^ Copyright 2011, Wiley‐VCH. d) Molecular structures of **154** and **155**, and corresponding electrochemical transformation reaction mechanism. Reproduced with permission.^[^ ^55^ ^]^ Copyright 2021, Wiley‐VCH.

Bis‐pyridinium polyenes can be considered prototypes of molecular wires facilitating electron flow,^[^ ^72^ ^]^ while push‐pull polyenes exhibit pronounced non‐linear optical properties.^[^ ^73^ ^]^ Combining these components with an externally triggered switching process could lead to electro‐photo or opto‐photo switches. Branda et al.^[^ ^74^ ^]^ discovered that the electrochemical reduction of the open‐state dicationic bispyridinium DTE **152** results in cyclization, producing the same ring‐closed isomer as the photochemically generated structure (**Figure** **18**a). Unexpectedly, a previously unobserved ring‐closed compound, *cis*‐**152**c,^[^ ^75^, ^76^ ^]^ was observed, which can only result from the disrotatory pericyclic reaction of the parallel conformer **152**o‐p. Although the **152**o‐p conformer coexists in almost equal proportion to the antiparallel conformer **152**o‐ap, only the photoexcited **152**o‐ap converts to its ring‐closed form *trans*‐**152**c (Figure 18a). They proposed that the electrochemical ring‐closing reaction proceeds via radical intermediates, avoiding the limitations of the photochemical reaction and leading to the formation of *cis*‐**152**c. In further investigation of **152**, Cobo et al.^[^ ^77^ ^]^ found that chemical reactions associated with the redox processes led to the isolation and characterization of a new DTE derivative (Figure 18b) incorporating a seven‐membered ring (7‐DTE), although these compounds are not stable over time.

Similar reductive cyclization was observed for dicationic DAE **153** ^[^ ^78^ ^]^ with thiazolpyridine aryl groups (Figure 18c). Carefully electrochemical studies, coupled with EPR spectroscopy, showed that this reductive cyclization occurs when **153** is reduced by two electrons. The same group then investigated terthiazoles with redox‐active substituents like an N‐methyl pyridinium group and ferrocene, resulting in DAEs **154^2+^** and **155^2+^**.^[^ ^55^ ^]^ The presence of two lateral N‐methyl pyridinium substituents effectively induces both reductive cyclization and oxidative cycloreversion, leading to the first terylene‐based switch capable of full photochemical and electrochemical operation (Figure 18d). However, introducing a second redox‐active unit, such as ferrocene, onto the central thiazolyl moiety inhibits photochromism but improves electrochromic cycloreversion via the redox properties of the ferrocenyl unit. The optical and redox properties of the switch in its different oxidation states were analyzed with the aid of DFT calculations to rationalize the various switching processes.

#### 2.3.4 Photoelectrocatalytic Electrochromism

**Figure 19:**
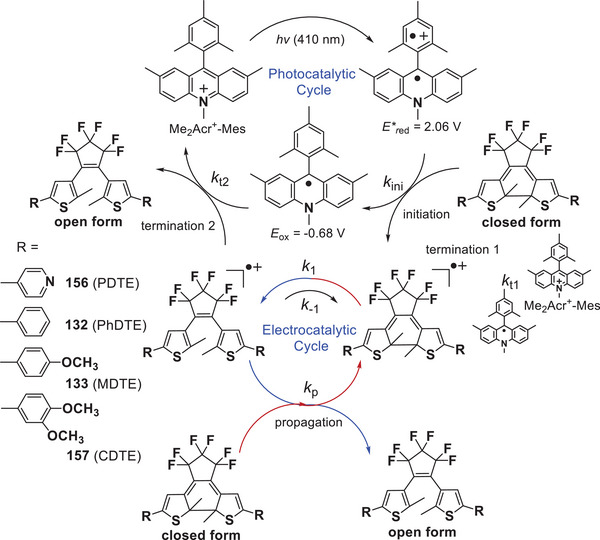
Mechanism of the photoelectrocatalytic cycloreversion of DTEs with photoredox catalyst Acr^+^‐Mes. Reproduced with permission.^[^ ^81^ ^]^ Copyright 2012, Wiley‐VCH.

The electrochromic process involves radical intermediates with significantly lower activation energies. The photoredox catalyst 9‐mesityl‐10‐methylacridinium ion (Acr^+^‐Mes) can transiently generate a long lifetime (e.g., 2 h at 203 K), high energy electron‐transfer (eT) state (Acr^•^‐Mes^•+^, 2.37 eV) upon photoexcitation,^[^ ^79^, ^80^ ^]^ effectively oxidizing certain organic molecules to radical cations. It is expected that combining DAEs and Acr^+^‐Mes would facilitate a new type of electrochromism initiated by photoexcitation without anoxidant. Inspired by this hypothesis, You et al.^[^ ^81^ ^]^ developed a photoelectrocatalytic strategy to achieve cycloreversion with high fatigue resistance over repeated chromic cycles. They previously demonstrated that four DTEs (PDTE **156**, PhDTE **132**, MDTE **133**, and CDTE **157**) undergo cycloreversion upon exposure to one‐electron oxidants such as Cu(ClO_4_)_2_, Fe(bpy)_3_](PF_6_)_3_, and [Ru(bpy)_3_](ClO_4_)_3_.^[^ ^57^ ^]^ When combined with Acr^+^‐Mes, these DTEs undergo photoelectrocatalytic cycloreversion. As illustrated in **Figure** **19**, an exoergic eT from DTE(c) to the Mes^•+^ moiety of Acr^•^‐Mes^•+^ initiates the electrochromism of DTE(c), competing with the back electron transfer (BeT) from the Acr^•^ moiety to DTE(c)^•+^ (termination 1). The eT from DTE(c) to the DTE(o)^•+^ completes cycloreversion, regenerating DTE(c)^•+^ and representing the propagation step in the electrocatalytic chain mechanism. This process terminates with BeT from the Acr^•^ moiety to the DTE(o)^•+^ (termination 2). Unlike photon‐stoichiometric photochromism, this process yields a leverage effect, significantly enhancing photochromic quantum yields (*Φ*_c‐o_) up to 0.54.

**Figure 20:**
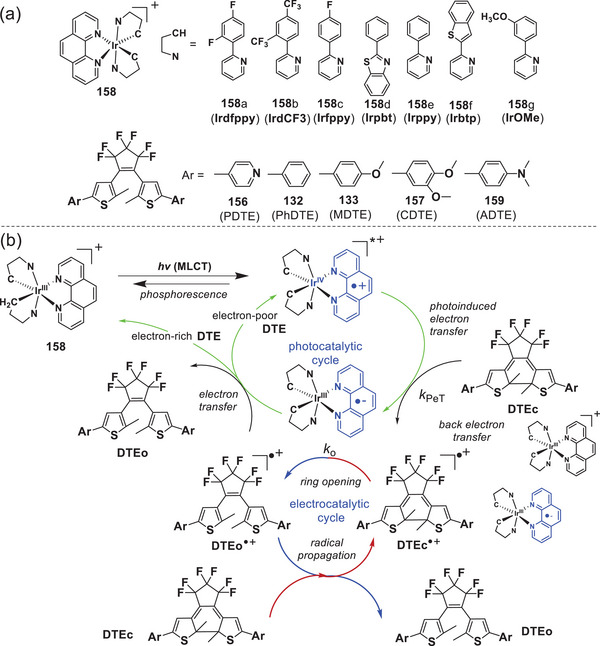
a) Molecular structures of cyclo‐metallated Ir(III) complexes and DTEs; b) proposed mechanism for the photoelectrocatalytic cycloreversion of DTEc compounds by Ir(III) complexes as the photoredox catalysts. Reproduced with permission.^[^ ^82^ ^]^ Copyright 2014, Royal Society of Chemistry.

To verify the suitability of photoredox catalyst in photo electrocatalytic cycloreversion and identify factors that ultimately improve the *Φ*_c‐o_, the same group^[^ ^82^ ^]^ employed seven cyclo‐metalated Ir(III) complexes (**Figure** **20**a) as visible light‐driven photoredox catalysts. Photon absorption by the ^1^MLCT transition of the Ir(III) complex [Ir^III^(C^N)_2_phen]^+^ promotes the electronic transition from the *d*‐orbital of the Ir(III) to the π*‐orbital of the ligand (i.e.,^1^[Ir^IV^(C^N)(C^N)^•−^phen]*^+^ or ^1^[Ir^IV^(C^N)_2_phen^•−^]*^+^). Subsequent intersystem crossing affords the triplet MLCT (^3^MLCT) transition state (i.e., ^3^[Ir^IV^(C^N)(C^N)^•−^phen]*^+^ or ^3^[Ir^IV^(C^N)_2_phen^•−^]*^+^), responsible for the oxidation of DTE(c). Figure 20b summarizes the proposed mechanism, comprising three key steps: (1) generation of DTE(c)^•+^, (2) cycloreversion of DTE(c)^•+^ to DTE(o)^•+^, and (3) reductive termination to DTE(o), with the generation of the ground or excited state of the catalyst. Ultimately, a quantitative *Φ*_c‐o_ was achieved with an enhancement of one order of magnitude relative to the controls (i.e., *Φ*_c‐o_ of PDTE is 0.38 with IrOMe compared to 0.016 without the photoredox catalyst). These findings offer valuable guidance for designing truly reversible photochromism and its applications in a wide range of molecular photonic systems.

## 3 Conclusion and Outlook

The introduction of stimuli beyond light to drive the cyclization and cycloreversion of DAE switches has achieved multi‐response and multi‐functional molecules and led to diverse potential applications, including energy converting, logic gates, and electronic and optical devices. The past two decades have witnessed the significant growth of this field. This review systematically examines the reaction mechanisms from three perspectives: acidochromism, thermochromism, and electrochromism, and highlights the recent advancements.

Despite substantial progress in enriching molecular structure diversity, challenges remain:

1. Current acid stimuli involve complex molecular structures and external chemicals, only quinone derivatives were developed as the ethene bridge of DAE. Developing a broader variety of molecules with this functionality remains a significant challenge. Given the essential role of the quinone moiety for DAEs **1**–**12** in the acid‐induced cyclization reaction, it is proposed that incorporating a quinone group into the side position of armed thiophene could similarly impart acid‐triggered cyclization characteristics to DAEs. Alternatively, replacing thiophene with benzothiophene may also achieve this effect.
2. For thermodynamic control of DAE switching, all known examples currently achieved are limited to the cycloreversion process. The thermal reaction generated by closed‐ring isomers is not observed for common DAEs. Developing DAEs with thermodynamic control over cyclization processes remains a significant challenge in this field. The complexity is heightened when attempting to achieve simultaneous thermodynamic regulation of both cycloreversion and cyclization. In cases where cyclization is thermally activated, cycloreversion would theoretically require even lower activation energy, implying a transition state that is accessible at a lower temperature than that of cyclization, which is impractical.
3. In electrochemical switching, the redox behavior of DAEs is significantly influenced by the molecular skeleton and substitution pattern. Modifying the side aryl groups and ethene bridge in DAEs can yield diverse electrochemical responses, including bidirectional reactions. On the other hand, combining photochromism, acidochromism, and thermochromism can achieve multi‐responsive DAE systems. For instance, photo‐ and electro‐dual‐switching molecules, which enable efficient photochemical cyclization and electrochemical cycloreversion, present great potential as active materials for optical writing/electric erasing nonvolatile memory, and future in‐situ fine image display. Additionally, the development of solid‐state electrolyte matrices, such as conductive gels and ionic liquid polymers, should be prioritized to enhance their applicability in future technologies.

We believe that with collective efforts, DAEs' response to beyond‐light stimuli for switching will exhibit promising prospects.

## Conflict of Interest

The authors declare no conflict of interest.
